# *BRCA2* N372H Polymorphism and Risk of Epithelial Ovarian Cancer: An Updated Meta-Analysis With 2344 Cases and 9672 Controls

**DOI:** 10.1097/MD.0000000000001695

**Published:** 2015-10-23

**Authors:** Liangxiang Su, Jing Wang, Yumei Tao, Xuefeng Shao, Yiqian Ding, Xiaoyan Cheng, Ying Zhu

**Affiliations:** From the Clinical Laboratory in Nantong Maternity and Child Health Care Hospital (LS, JW, XS, YZ); Pathology Department of Nantong Maternity and Child Health Care Hospital (YT); and Department of Gynaecology and Obstetrics of Nantong Maternity and Child Health Care Hospital, Nantong, Jiangsu Province, China (YD, XC).

## Abstract

The *BRCA2* N372H is the only common polymorphism that leads to the amino acid change based on the reports up to date. Previous studies explored the relationship between the single nucleotide polymorphism and ovarian cancer risk, but the results were inconsistent or inconclusive.

To investigate the association between N372H in *BRCA2* gene and ovarian cancer susceptibility, a systematic literature search was performed for related publications in the databases of PubMed, Gene, and Google Scholar.

Total 2344 cases and 9672 controls in eligible studies were included in this meta-analysis. χ^2^ -based Q test and an I^2^ index were used to identify the heterogeneous records. Potential publication biases were assessed by Begg and Egger tests.

In the overall analysis, the results showed a significant association between *BRCA2* codon 372 polymorphism and increased risk of ovarian cancer (HH versus NN: odds ratio (OR) = 1.22, 95% confidence interval (CI) 1.01–1.48, *P* = 0.037). In the Australia subgroup analysis, significant association was also detected (HH versus NN: OR = 1.40, 95% CI 1.04–1.87, *P* = 0.026). The subgroup analysis for serous cancer subgroup showed that the significant association could be detected under recessive model (OR = 1.38, 95% CI, 1.01–1.89, *P* = 0.04) and under homozygote comparison (OR = 1.46, 95% CI, 1.06–2.01, *P* = 0.022).

Our meta-analysis suggests that the N372H polymorphism is associated with susceptibility of ovarian cancer. The allele H might increase the risk of ovarian cancer, especially, for ovarian cancers of the serous subtype.

## INTRODUCTION

Ovarian cancer is the most lethal gynecologic malignancies worldwide. The vast majority of malignant ovarian cancers is of epithelial origin and can be classified into 4 major subtypes: serous, mucinous, endometrioid, and clear cell.^1^ Among them, serous epithelial ovarian cancer is the most common subtype, accounts for about 70% of all ovarian cancer cases diagnosed. Despite the intensive studies in this field, which profoundly advanced our understanding of ovarian cancer, the exact molecular pathogenesis of epithelial ovarian serous carcinoma still remains elusive.^2^ And the traditional screening strategies, such as ultrasound and CA-125 level detection have not yet been shown to improve overall survival.^3^

*BRCA2* involves in DNA double-strand break repair pathway. The mutations on this gene confer high susceptibility to ovarian cancer.^4^ Although recent studies already identified several high-risk ovarian cancer genes, common low-penetrance susceptibility alleles might still exist, which leads to moderate increase in ovarian cancer risk. Among all the single nucleotide polymorphisms, the rs144848 in exon 10 is the only polymorphism that change the amino acid (from asparagine to histidine), with an exceptional high-allele frequency.^5^ The transition is located at the region (residues 290–453), which has been identified to interact with the histone acetyltranferase P/CAF.^6^

Compared with the many published studies on this polymorphism for breast cancer susceptibility,^7–14^ only a few studies focused on this single nucleotide polymorphism for ovarian cancer risk, and the conclusions are inconsistent. We thus, performed this meta-analysis to derive a more precise and up-to-date estimation of the association between *BRCA2* N372 and ovarian cancer risk.

## MATERIAL AND METHODS

### Data Collection

Literature search was performed from the NCBI Global Cross-database, including PubMed, Gene, as well as Google Scholar with the following keywords: “*BRCA2* N372H polymorphism,” “rs144848,” “*BRCA2* Asn372His polymorphism,” and “ovarian cancer.”

To obtain the qualified data for this analysis, we set up the following inclusion criteria: the data should come from case-control studies; the articles should aim at investigating the association between *BRCA2* N372H polymorphisms and ovarian cancer risk; the raw data should have sample size, odds ratio (OR) with 95% confidence interval (CI), or the relevant information should be able to be retrieved; if the studies from different articles overlap, we only keep the ones showing the most extensive results.

**FIGURE 1:**
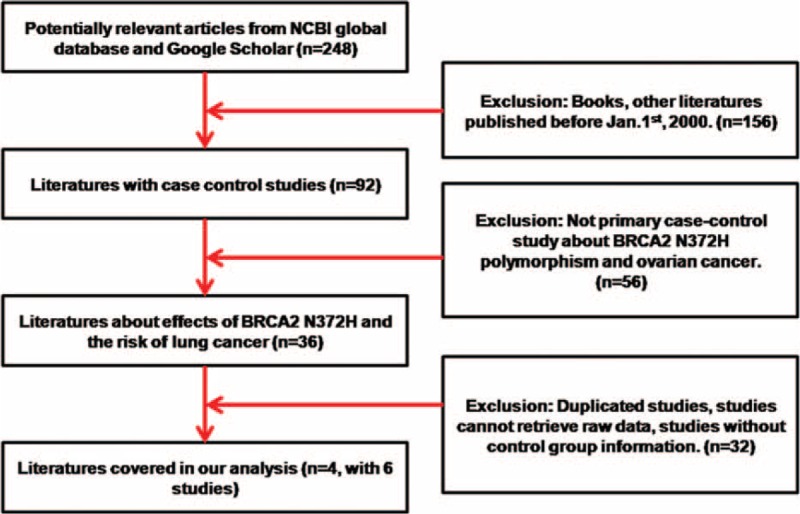
Data collection procedure.

After a thorough review of all articles from the literature search, total 6 case-control studies from 4 articles were included in our final meta-analysis for association between N372H polymorphisms and ovarian cancer risk. Based on the Newcastle–Ottawa Scale^15^ evaluation, all studies were evaluated as well-defined and eligible to be referred to in a meta-analysis (adequately defined case/control group, good representativeness of case/control group, good comparability between case, and control group). The data collection flow chart is shown in Figure 1.

Our analyses were based on previous published studies, thus, no ethical approval and patient consent are included.

### Data Extraction

We extracted the following information from all qualified studies: first author, publication date, countries, ovarian cancer subtypes, number of cases, and controls and the frequencies of *BRCA2* codon 372 polymorphisms in both cases and controls. Characteristics of individual studies were summarized in Table 1.

**TABLE 1:**
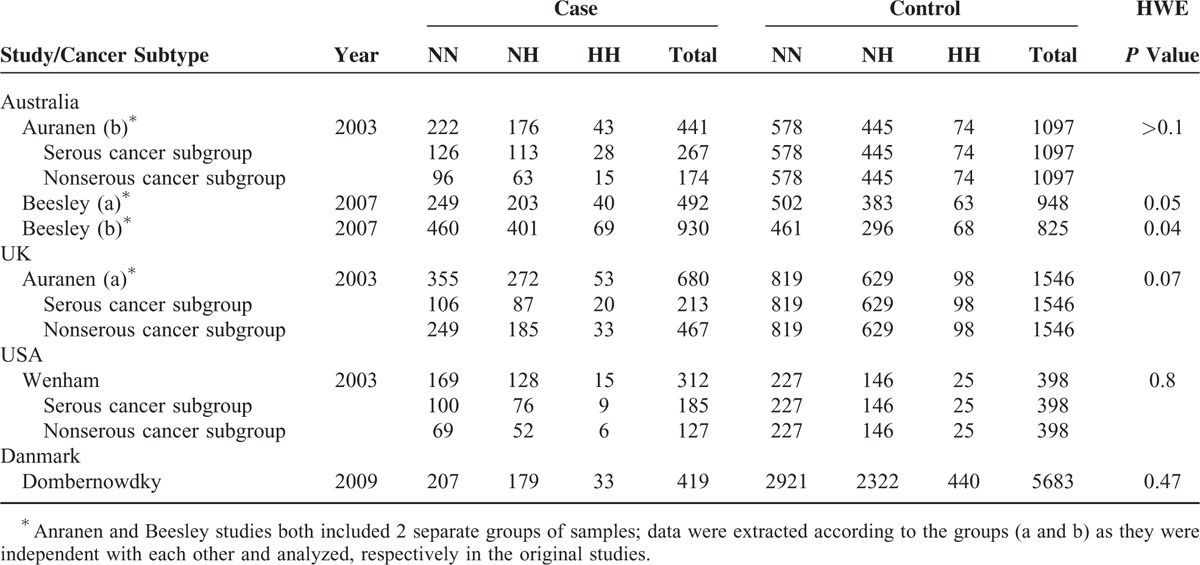
Pooled Data for *BRCA2* N372H Polymorphism Analysis

### Statistical Analysis

The genotype data were subjected to asymptotic Pearson χ^2^ test for the goodness of fit of Hardy–Weinberg Equilibrium in controls. A *P*-value <0.05 was considered as significant disequilibrium. The results were shown in Table 1. The OR and corresponding 95% CI for alleles and genotypes were used to calculate the association between *BRCA2* gene polymorphism and ovarian cancer susceptibility from the extracted dataset. The heterogeneity between studies was examined by χ^2^-based Q test and I-squared index. When there was no significant heterogeneity (*P*-value >0.1 and I^2^ < 50%), the pooled OR was estimated by Mantel–Haenszel fixed-effect model.^16^ Otherwise the D-L random-effect model ^17^ was applied. The risk of dominant model (NH + HH versus NN), recessive model (HH versus NH + NN), and homozygote model (HH versus NN) of *BRCA2* gene polymorphism for the entire dataset were evaluated, respectively with ORs and 95% CIs. Subgroup analysis based on ethnic (covering 3 studies from Australia) or different cancer subtypes (serous ovarian cancer versus nonserous ovarian cancer) were carried out similarly. Meta-analysis was performed by STATA (version 12.0). Publication bias was assessed by Begg funnel plot and Egger linear regression test. The asymmetric funnel plot is an indication of possible publication bias.

## RESULTS

### Subject Characteristics

A total of 4 articles (including 6 studies) from 248 publications were included in present meta-analysis.^18–21^ Detailed exclusion criteria were shown in Figure 1 for unqualified publications. All the data in these included studies were related to the association between *BRCA2* N372H polymorphism and human ovarian cancer risk. The Hardy–Weinberg Equilibrium for all 4 publications was calculated. One of the 2 datasets from Beesley study (b) was excluded from the final analysis because of the statistically significant deviation from HWE (*P* value = 0.04). No significant violation of HWE (all P values greater than 0.05) was detected for other 5 studies including Auranen (a), Auranen (b), Beesley (a), Wenham and Dombernowdky. The characteristics of all studies with 2344 cases and 9672 controls were summarized in Table 1.

### Meta-Analysis

**FIGURE 2:**
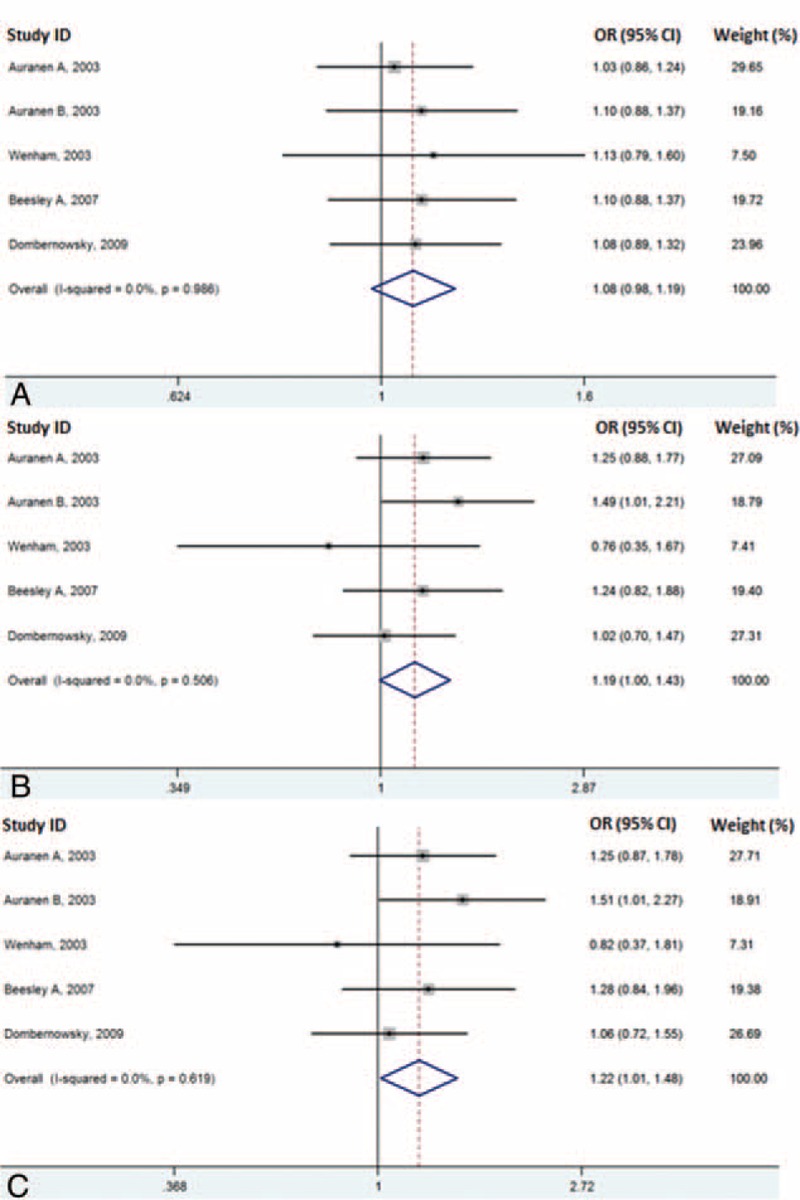
Risk evaluation of *BRCA2* codon 372 polymorphism for entire database under the (A) dominant model (NH + HH versus NN), (B) recessive model (HH versus NH + NN), and (C) homozygote model (HH versus NN).

In the overall analysis, we found a significant strong association between *BRCA2* N372H polymorphism and increased risk of ovarian cancer in the homozygote model (HH versus NN) with OR 1.22 (95% CI, 1.01–1.48, *P* = 0.037) (Table 2, Fig. 2C). No statically significant association could be detected under the dominant model (NH + HH versus NN) with OR 1.08 (95% CI, 0.98–1.19, *P* = 0.134) and recessive model (HH versus NN + NH) with OR 1.20 (95% CI, 1.00–1.43, *P* = 0.056) (Table 2, Figs. 2A-B).

**TABLE 2:**
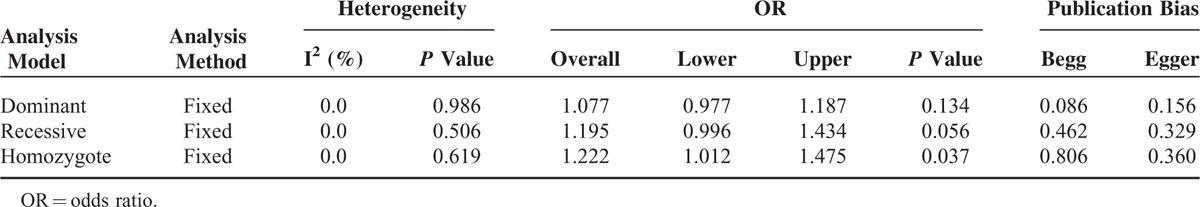
Meta-Analysis for Entire Database With Dominant Model (NH + HH Versus NN), Recessive Model (HH Versus NH + NN), and Homozygote Model (HH Versus NN)

In the subgroup analysis for the Australia group, we also detected a significant strong association under the homozygote model (HH versus NN) with OR 1.40 (95% CI, 1.04–1.87, *P* = 0.026) (Table 3, Fig. 3 C) and recessive model (HH versus NN + NH) with OR 1.67 (95% CI, 1.03–1.82, *P* = 0.032) (Fig. 3 B). For dominant model, the overall OR was 1.10 (95% CI, 0.94–1.28, *P* = 0.235) (Table 3, Fig. 3 A).

**TABLE 3:**
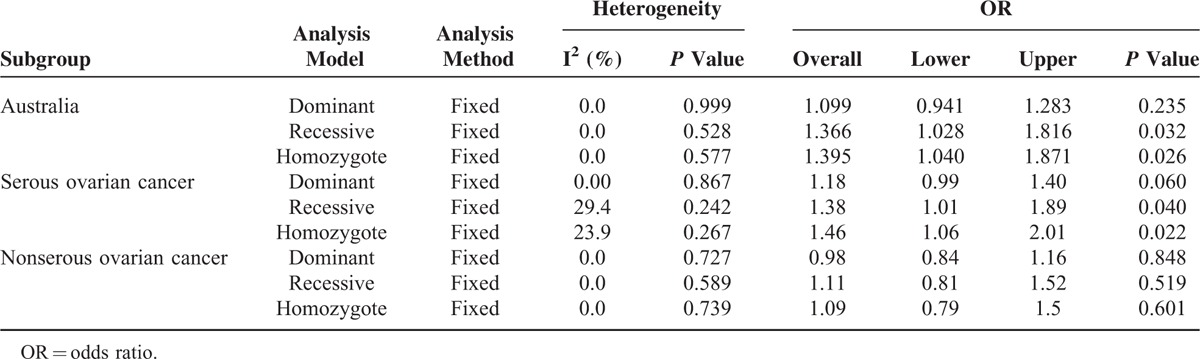
Subgroup Meta-Analysis With Dominant Model (NH + HH Versus NN), Recessive Model (HH Versus NH + NN), and Homozygote Model (HH Versus NN) for Australia, Serous Ovarian Cancer, and Nonserous Ovarian Cancer

**FIGURE 3:**
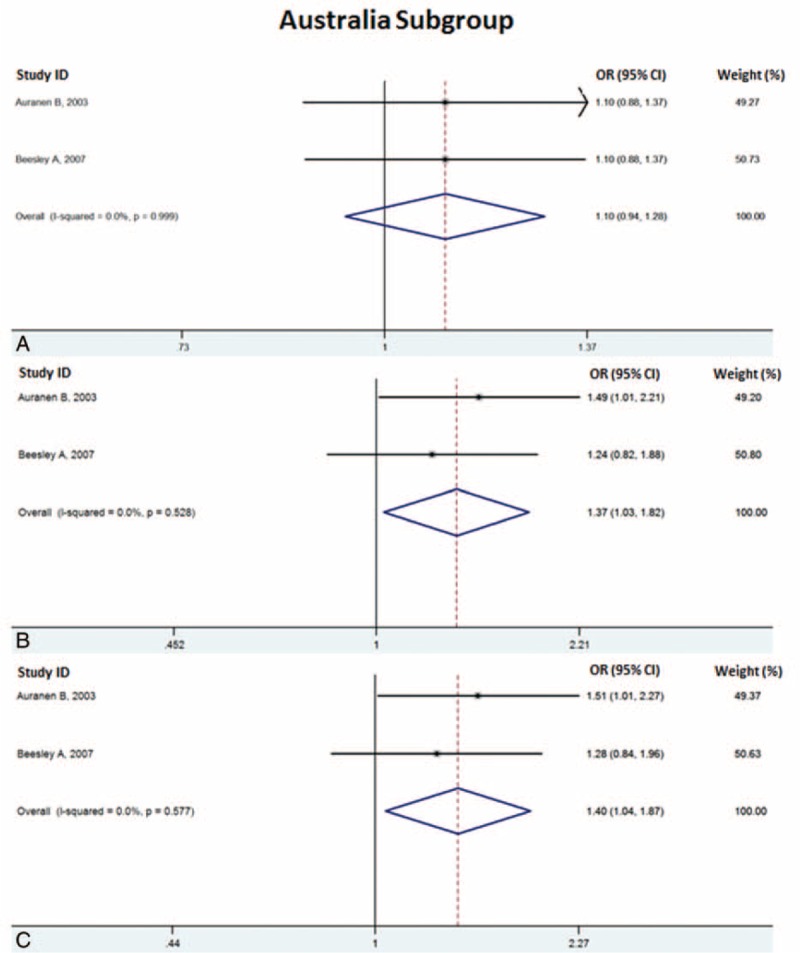
Risk evaluation of *BRCA2* codon 372 polymorphism under the (A, D, and G) dominant model (NH + HH versus NN) (B, E, and H), recessive model (HH versus NH + NN), and (C, F, and I) homozygote model (HH versus NN) for Australia subgroup, serous cancer subgroup, and nonserous cancer subgroup.

**FIGURE 3 (Continued):**
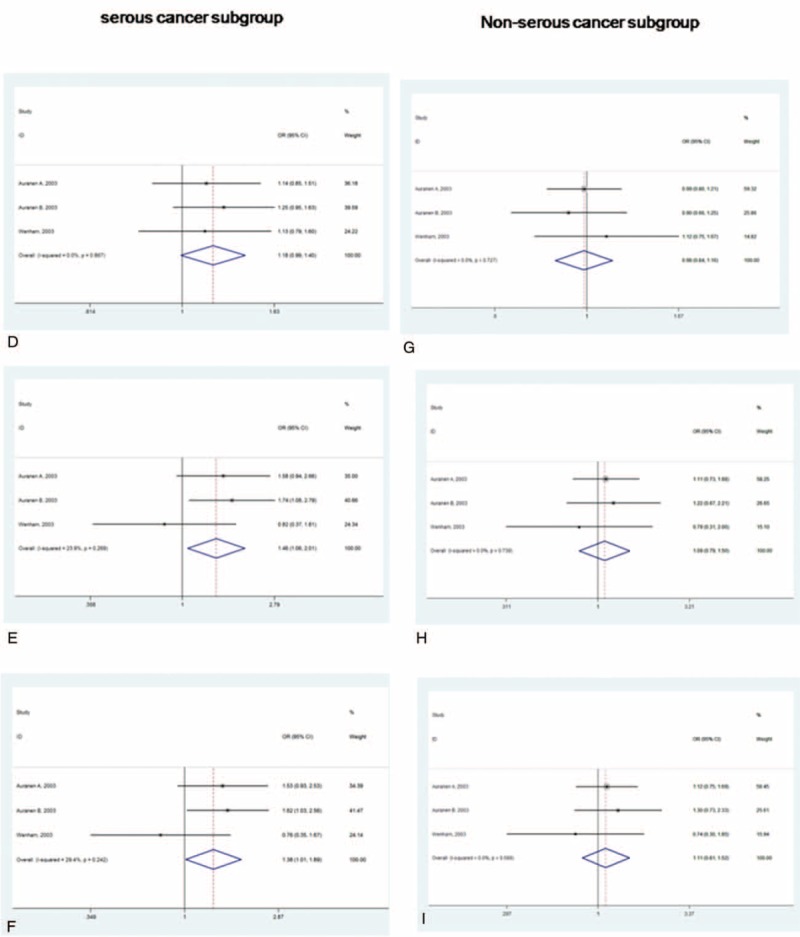
Risk evaluation of *BRCA2* codon 372 polymorphism under the (A, D, and G) dominant model (NH + HH versus NN) (B, E, and H), recessive model (HH versus NH + NN), and (C, F, and I) homozygote model (HH versus NN) for Australia subgroup, serous cancer subgroup, and nonserous cancer subgroup.

The subgroup analysis for serous cancer subgroup showed that the *BRCA2* N372H polymorphism has marginally elevated risk in the dominant model with the overall OR 1.18 (95% CI, 0.99–1.40, *P* = 0.06). Statically significant association could be detected under recessive model (OR was 1.38, 95% CI, 1.01–1.89, *P* = 0.04) and under homozygote comparison (OR was 1.46, 95% CI, 1.06–2.01, *P* = 0.022) (Table 3, Figs. 3 D–F). On the contrary, no difference was observed between case and control group in nonserous subtype subgroup. For dominant model, the overall OR was 0.98 (95% CI, 0.84–1.16, *P* = 0.848). For recessive model, the overall OR was 1.11 (95% CI, 0.81–1.52, *P* = 0.519) and for homozygote comparison, the overall OR was 1.09 (95% CI, 0.79–1.50, *P* = 0.601) (Table 3, Figs. 3 G–I).

### Evaluation of Heterogeneity

When we examined the heterogeneity for the entire dataset under all 3 allele comparison models, the *P*-values by χ^2^-based Q test were all greater than 0.1 and I^2^ indexes were all smaller than 50% (Table 2), indicating no statistically significant heterogeneity between studies. Similarly no significant heterogeneity was observed under all 3 models when we performed subgroup analysis for Austria dataset and different cancer subtype dataset (Table 3).

### Potential Publication Bias

**FIGURE 4:**
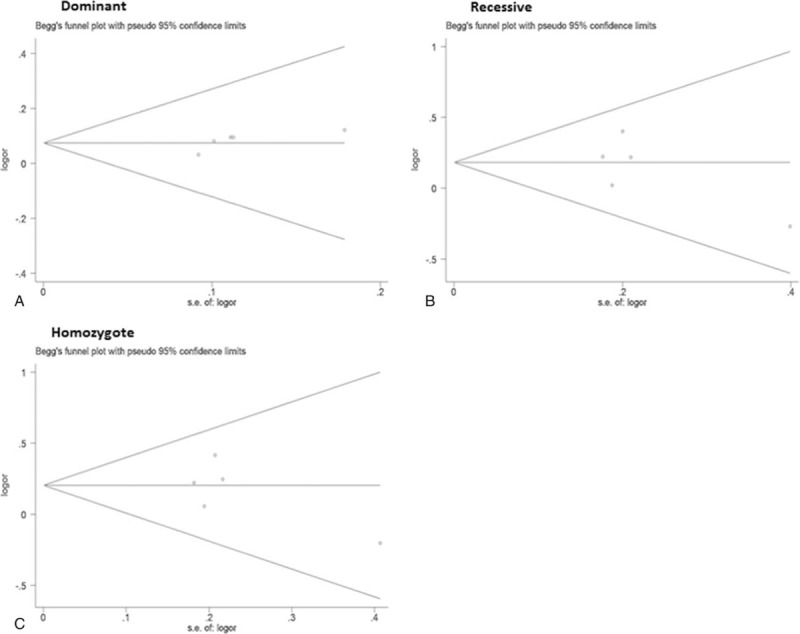
Begg funnel plot with pseudo 95% confidence limits for BRCA codon 372 polymorphism for entire database under different models. A, Dominant model (NH + HH versus NN). B, Recessive model (HH versus NH + NN). C, Homozygote model (HH versus NN).

We examined the potential publication bias for the overall datasets by Begg funnel plots and Egger linear regression analysis. All the funnel plots under all 3 models were symmetrical (Fig. 4) and the *P*-values from Egger test were all great than 0.05 (Table 2). Meanwhile, funnel plot and Egger test were not available for all subgroup datasets because of small sample size.

## DISCUSSION

Several genes polymorphisms in ovarian cancer risk have been explored, such as Vitamin D receptor FokI,^22^ human epididymis protein 4 (HE4),^23^ the progesterone receptor,^24^ p53 ^25–27^, and RAD52.^28^ In our study, we investigated the association of *BRCA2* gene N372H polymorphism with ovarian cancer risk in the context of a case-control study. The majority of ovarian cancers are sporadic; only approximately 10% cases are hereditary. Although mutations on *BRCA2* is one of the major cause of hereditary ovarian cancers, the expression profiles are also altered in sporadic ovarian cancers, which are known as the “BRCAness”of sporadic cancers. Therefore, it is logical to seek low-penetrance susceptibility alleles in this gene. Among all the *BRCA2* gene SNPs reported up-to-date, N372H **(**rs144848) is the most common polymorphism that leads to an amino acid change, where the basic N residue is substituted by the structurally smaller neutral residue H. Although the exact function of this nonconservative substitution is still unknown, it might alter the protein structure and function, as it is located at the N-terminal region where *BRCA2* interacts with P/CAF to activate other gene transcription.

Previous studies related to N372H polymorphism and ovarian cancer risk were either based on relative smaller sample size, different ethnic group, or different methodologies. The conclusions were somehow inconsistent or inconclusive. For example, Auranen et al (2003) found that the HH genotype was associated with an increased risk of ovarian cancer with OR 1.36 (95% CI 1.04–1.77, *P* = 0.03) based on 1121 ovarian cancer cases and 2643 controls from British, and Australian studies. Although Wenham et al (2003) concluded that no such association could be detected in their study from 312 cases and 401 controls in North Carolina. In the present meta-analysis, we did not find a statistically significant association under the homozygous model (HH versus NN) for either overall or Australia subgroup data from pooled 3147 cases and 10,497 controls. From the combined analysis of all 5 studies, the genotypes (NH + HH), however, were determined to have a 1.12× increased risk (95% CI, 1.03–1.22, *P* = 0.01) for ovarian cancer compared with 372NN genotype. For the Australia subgroup analysis, we also detected a significant association between N372H polymorphism and ovarian cancer risk under the dominant model (NH + HH versus NN) with OR = 1.17 (95% CI, 1.04–1.32, and *P*-value = 0.009). These results suggested that the allele H at BRAC2 codon 372 has a moderate yet definite genetic effect. The genotypes NH and HH carriers have an increased risk of ovarian cancer.

The present meta-analysis also indicated that there exists a much stronger association between *BRCA2* N372H polymorphism with ovarian cancers of the serous subtype compared with other ovarian cancer subtypes. As in the subgroup analysis for serous ovarian cancer, our study showed that the risk was even higher for the HH homozygotes (1.38× to 1.42× increased risk). This is in agreement with other previous studies, as they also suggested the HH homozygotes may be at greater risk in serous ovarian cancers.^18^

Meanwhile, some limitations might be present in our analysis: The heterogeneity between studies—we examined the heterogeneity in present analysis by χ^2^-based Q test and I squared test. The *P*-values were >0.1 and I^2^ were <50% for all the analysis performed in our study, indicating that no significant heterogeneity was detected; Publication bias—in our study, potential publication bias was visualized by the funnel plot and was further evaluated by Egger test. The *P-*values of Egger test are greater than 0.5 under all models for overall datasets, which provided evidence for the symmetry of funnel plots in our meta-analysis. We cannot, however, exclude the possibility of publication bias for Australia subgroup dataset, as only 3 publications were included in this subgroup. Caution should be taken when interpreting the results in this subgroup. More data are required to generalize our results in other ethnicities. HWE test in the control group showed that the distribution of genotypes in 1 of the 2 datasets from Beesley study is not in HWE, possibly because of systematic errors in genotyping or others. As for the retrospective study, meta-analysis is subject to methodological limitations, which emphasizes the need to interpret our results with caution.

In conclusion, our meta-analysis suggests that the N372H polymorphism is associated with susceptibility of ovarian cancer. The allele H has a moderate yet definite genetic effect. The risk may be even greater for ovarian cancers of the serous subtype.
